# The mitochondrial DNA 4,977-bp deletion and its implication in copy number alteration in colorectal cancer

**DOI:** 10.1186/1471-2350-12-8

**Published:** 2011-01-13

**Authors:** Tao Chen, Jing He, Lijun Shen, Hezhi Fang, Hezhongrong Nie, Tao Jin, Xiaosong Wei, Yijuan Xin, Yulin Jiang, Hongzhi Li, Guorong Chen, Jianxin Lu, Yidong Bai

**Affiliations:** 1 Zhejiang Provincial Key Laboratory of Medical Genetics, School of Laboratory Medicine of Wenzhou Medical College, Zhejiang 325035, PRChina; 2 Department of Pathology of the First Affiliated Hospital, Wenzhou Medical College, Wenzhou 325000, PR China; 3 Department of Cellular and Structural Biology, University of Texas Health Science Center at San Antonio, San Antonio, TX 78229, USA

## Abstract

**Background:**

Qualitative and quantitative changes in human mitochondrial DNA (mtDNA) have been implicated in various cancer types. A 4,977 bp deletion in the major arch of the mitochondrial genome is one of the most common mutations associated with a variety of human diseases and aging.

**Methods:**

We conducted a comprehensive study on clinical features and mtDNA of 104 colorectal cancer patients in the Wenzhou area of China. In particular, using a quantitative real time PCR method, we analyzed the 4,977 bp deletion and mtDNA content in tumor tissues and paired non-tumor areas from these patients.

**Results:**

We found that the 4,977 bp deletion was more likely to be present in patients of younger age (≤65 years, p = 0.027). In patients with the 4,977 bp deletion, the deletion level decreased as the cancer stage advanced (p = 0.031). Moreover, mtDNA copy number in tumor tissues of patients with this deletion increased, both compared with that in adjacent non-tumor tissues and with in tumors of patients without the deletion. Such mtDNA content increase correlated with the levels of the 4,977 bp deletion and with cancer stage (p < 0.001).

**Conclusions:**

Our study indicates that the mtDNA 4,977 bp deletion may play a role in the early stage of colorectal cancer, and it is also implicated in alteration of mtDNA content in cancer cells.

## Background

Colorectal cancer is one of the leading human malignancies [1]. While its morbidity and mortality have declined in western countries in recent years, both the incidence and deaths caused by this cancer have increased significantly in recent years in Asia [2], particularly in China [3]. Both genetic and environmental factors contribute to colorectal cancer development. Based on a study on cohorts of twins from Sweden, Denmark and Finland, heritable factors contributed about 35% to colorectal cancer [4], while in another nationwide family study conducted with 9.6 million Swedish people, around 13% of colorectal susceptibility was attributed to genetic effects [5]. However, up to now, only 6% of colorectal cancer can be ascribed to mutations in particular genes [6]. Among those genes associated with predispositions for colorectal cancer are adenomatous polyposis coli (APC), a tumor suppressor involving cell adhesion, signal transduction and transcription activation, and DNA mismatch repair (MMR) genes [6,7].

Mitochondria, known as the cellular power plants, also regulate cell death and cell proliferation [8,9]. Defects in mitochondrial function have long been hypothesized to play a role in tumorigenesis [10]. Mitochondria possess their own genomes. Human mtDNA encodes 13 essential subunits of the oxidative phosphorylation (OXPHOS) system as well as 2 rRNAs and 22 tRNAs used for mitochondrial translation [11]. Alterations in mtDNA both qualitatively (mutations) [12-14] and quantitatively (mtDNA copy number) [15] have been associated with many human diseases including neurodegenerative diseases, metabolic diseases and various types of cancer [16-18].

The mtDNA is subject to relatively high oxidative damage and at the same time is sensitive to such damage. It has also been shown that oxidative modified DNA is especially prone to mispairing of repetitive elements and is correlated with deletions [19]. In fact, large scale deletions were among the first mtDNA mutations identified to cause human diseases [20,21]. Up to now, more than 100 deletions have been reported to be associated with various diseases (http://www.mitomap.org/). Among these deletions, a 4,977-bp deletion occurring between two 13-bp direct repeats at positions 13447-13459 and 8470-8482 has attracted tremendous interests since it is the common cause of several sporadic diseases including Pearson's syndrome, Kearns-Sayre syndrome (KSS) and chronic progressive external ophthalmoplegia (CPEO) [13,22], and is therefore called the "common" deletion. This deletion also accumulates in many tissues during aging, and has been used as an mtDNA damage biomarker [18,23].

mtDNA mutations, including both point mutations and deletions, have been identified in various types of human cancer [16,17,24]. In one of the first comprehensive studies of mtDNA in cancer cells, it was demonstrated that among ten colorectal cancer cell lines, seven of them exhibited mtDNA mutations [25]. In another study focused on the major control region of mtDNA, the "displacement loop" (D-loop) in French colorectal cancer patients, the presence of tumor D-loop mutations correlated with poor prognosis [26].

To address the question if the mtDNA 4,977-bp deletion plays a role in pathogenesis and development of colorectal cancer, we studied 104 colorectal cancer patients recently admitted in the First Affiliated Hospital of Wenzhou Medical College and carried out a systematic investigation into their clinicopathological features and characterized their mtDNA, in particular the 4,977-bp deletion and the mtDNA content, in the tumor tissues and nearby non-tumor areas.

## Methods

### Samples collection

Paraffin-embedded tumor tissues and paired adjacent non-tumor tissues were collected from 104 unrelated colorectal cancer patients prior to any chemotherapy, radiotherapy or pharmacotherapy at the First Affiliated Hospital of Wenzhou Medical College between October, 2006 and March, 2008. Informed consent from all patients in this study was obtained under protocols approved by the Wenzhou Medical College Ethics Committee. The patients ranged in age from 33 to 87 years (mean ± SD, 65.58 ± 11.49), and were classified according to the tumor-node-metastasis (TNM) staging system (American Joint Committee on Cancer): 15 were at stage I, 45 at stage II, 39 at stage III and 5 at stage IV. Ten-micron sections were cut from paraffin blocks and classifications were confirmed by a senior pathologist using a standard hematoxylin & eosin staining protocol.

### Detection of the mtDNA 4,977-bp deletion

**Figure 1:**
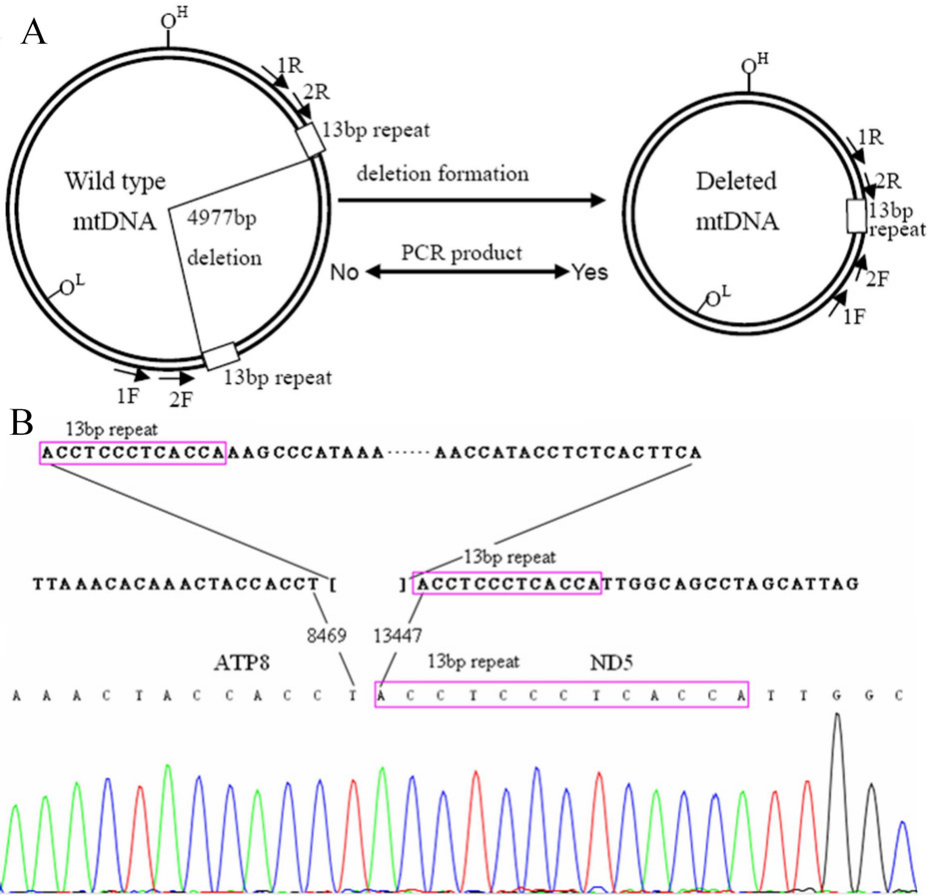
**Detection of the mtDNA 4,977-bp deletion**. (A) Human mitochondrial genome with or without the 4,977-bp deletion; the positions of primers used in nested PCR for detecting this deletion were 1F (8192-8212), 1R (13663-13643), 2F (8261-8281) and 2R (13595-13575). The fragment was too large for wildtype mtDNA (>5 kb) to be amplified with the PCR conditions used for detection. (B) Confirmation of the 4,977 bp deletion. Representative sequencing profile of the PCR products from the patients carrying the deletion.

Tumor and non-tumor tissues on the slides were extracted separately under a microscope. Genomic DNA was isolated as previously described [27]. To screen for the 4,977-bp deletion in mtDNA, nested PCR analysis was performed in order to detect low levels of deletion (Figure. 1). Two pairs of nested primers for detection of the 4,977-bp deletion were, 1F: AACCACAGTTTCATGCCCATC; 1R: TGTTAGTAAGGGTGGGGAAGC; 2F: ACCCTATTGCACCCCCTCTAC; and 2R: CTTGTCAGGGAGGTAGCGATG. The PCR condition was set as: pre-denaturation at 94°C for 5 min; then 30 cycles at 94°C for 10 s, 58 °C for 45 s and 72 °C for 50 s; and a final extension at 72 °C for 10 min. PCR products were then electrophoresed on a 2% agarose gel. By design (Figure. 1A), the presence of the 4,977-bp deletion was indicated by the appearance of a 358-bp band, which was verified by sequencing analysis (Figure. 1B). On the other hand, wild-type mtDNA as the template would not yield any PCR products under such conditions because of the large flanking region (>5-kb) (Figure. 1A). All PCR experiments included a negative control with no template DNA (double-distilled water) and a positive control with mtDNA harboring a 4,977-bp deletion which was isolated in the laboratory previously from a prostate cancer patient.

### Determination of mtDNA content and levels of the 4,977 bp deletion

The mtDNA content was measured by a real-time PCR on cytochrome c oxidase I (COX I) gene and normalized by simultaneous measurement of nuclear DNA encoded β -actin genes. QPCR was carried out using an ABI 7900HT Fast Real-Time PCR System (Applied Biosystems) in a 20 μl reaction in different tubes containing 0.5 μM each of the forward and reverse primers, 0.1 pM for each probe (COX I and β-actin genes) and 500 pg of DNA sample for mtDNA and 10 ng for nDNA. The PCR conditions were 95°C for 15 min, followed by 40 cycles of 95°C for 15 s, and 60°C for 60 s. The threshold cycle number (Ct) values of the β-actin gene and the mitochondrial COXI gene were determined.

The 4,977 bp deletion level was measured by real-time PCR on deletion product (Figure. 1) and normalized by simultaneous measurement of mtDNA COX I.

Each measurement was carried out in triplicate and normalized against a serial dilution of a control DNA sample and then the quantity of each target gene in our samples was calculated according to the corresponding standard curve.

The primer and probe information is as follows:

For mtDNA COX I: Forward, TTCGCCGACCGTTGACTATTCTCT; Reverse, AAGATTATTACAAATGCATGGGC.

For nuclear β-actin: Forward, ACCCACACTGTGCCCATCTAC; Reverse, TCGGTGAGGATCTTCATGAGGTA.

For mtDNA 4,977 bp deletion: Forward, CCTTACACTATTCCTCATCACC; Reverse, TGTGGTCTTTGGAGTAGAAACC

Probes: COX I, FAM-AACGACCACATCTACAACGTTATCGTCAC-ECLIPSE; β-actin, FAM-ATGCCCTCCCCCATGCCATCC-ECLIPSE. 4,977 -bp deletion, FAM-TGGCAGCCTAGCATTAGCAGG-ECLIPSE

Standard curves for deleted mtDNA were generated from a prostate cancer patient, and for COX I and β-actin genes they were obtained using a human osteosarcoma-derived cell line (U2OS) DNA. mtDNA content was calculated by dividing the amount of total mitochondrial DNA into the amount of nuclear gene. The mtDNA deletion level was expressed as the ratio of content of deleted mitochondrial DNA to total mtDNA content.

### Statistical analysis

Categorical variables were analyzed using chi-square test or Fisher's exact test and continuous variables were examined using Student's t-test. In order to adjust for the contribution of each clinicopathologic characteristic, logistic regression and linear regression analysis were also performed. All statistical results were calculated with SPSS 16.0 software and a p value less than 0.05 was considered statistically significant.

## Results

### Detection of the mtDNA 4,977 bp deletion in colorectal cancer patients

Of the 104 colorectal cancer patients, 20 (19.23%) showed the 4,977 -bp deletion in either tumor or nearby non-tumor tissues. Among them, 10 (9.62% of total) were found to be harboring the deletion in both tumor and non-tumor tissues, 7 (6.73%) only showed it in tumor tissues, and 3 (2.88%) only in non-tumor tissues. Overall, 17 patients were found to carry the 4,977-bp deletion in tumors, and 13 patients carried it in nearby non-tumor tissues (Table 1). We also analyzed some clinical characteristics and other risk factors for colorectal cancer in these patients including age, body mass index (BMI), tumor cell differentiation status, lymph node metastasis (LN metastasis) detection and tumor stage. As shown in Table 1, BMI seemed not to be a contributing factor for occurrence of the 4,977-bp deletion in either tumor or non-tumor tissues. Examination of the clinicopathologic features listed above failed to reveal any significant association between the occurrence of the 4,977-bp deletion and tumor cell differentiation, LN metastasis and tumor stage (Table 1). Surprisingly, unlike what was expected as the 4,977-bp deletion accumulated during aging, we found the patients under 65 years old were more likely to carry this deletion in tumor tissues. Although 12 out of 48 (25%) of colorectal cancer patients in this younger age group were found to harbor the 4,977-bp deletion in tumor tissues, only 5 of 56 (8.93%) older patients carried this deletion (p = 0.027) (Table 1). This age-related difference only existed in tumor tissues, and no differences were detected in the nearby non-tumor tissues (Table 1). Moreover, it appeared that in patients under 65, tumor tissues were more likely to carry the 4,977-bp deletion compared with the nearby non-tumor tissues (OR = 1.952; 95% CI: 0.694-5.491). In contrast, in the older age group, there was no such difference (OR = 0.817; 95% CI: 0.234-2.850). Interestingly, while the ages of 7 patients carrying 4,977-bp deletion only in tumor tissue were wide-ranging, all three patients with this deletion only in non-tumor areas were over 65.

**Table 1:** Detection of the mtDNA 4,977 bp deletion in tumor and non-tumor tissues of 104 colorectal cancer patients.

	N	Deletion detected in non-tumor tissues	p-value	OR (95% CI)	Deletion detected in tumor tissues	p-value	OR (95% CI)
Cases	104	13 (12.50%)			17 (16.35%)		
Age							
≤65	48	7 (14.58%)	0.552	1.423	12 (25.00%)	0.027*	3.400
>65	56	6 (10.71%)		(0.443-4.566)	5 (8.93%)		(1.101-10.495)
BMI#							
<18	3	0	0.248	--	0	0.286	--
18-24	65	8 (11.76%)			11 (16.18%)		
≥24	25	5 (20.00%)			6 (24.00%)		
Differentiation#							
Poor	22	2 (9.09%)	0.772	0.590	3 (13.64%)	0.813	0.680
Moderate	69	10 (14.49%)		(0.119-2.924)	13 (18.84%)		(0.175-2.647)
LN metastasis							
Positive	43	8 (18.60%)	0.114	2.560	8 (18.60%)	0.601	1.321
Negative	61	5 (8.20%)		(0.775-8.453)	9 (14.75%)		(0.465-3.753)
Stage							
I+II	60	5 (8.33%)	0.134	0.409	9 (15.00%)	0.665	0.794
III+IV	44	8 (18.18%)		(0.124-1.350)	8 (18.18%)		(0.280-2.255)

N, case number; BMI, body mass index; Differentiation, tumor cell differentiation status; LN metastasis, lymph node metastasis; Stage, cancer stage classified according to the tumor-node-metastasis (TNM) staging system (American Joint Committee on Cancer); OR, odds ratio; *P*-values were calculated by chi-square test, * statistically significant; 95% CI, 95% confidence interval; # incomplete data.

### Level of the 4,977 bp deletion and colorectal cancer development

**Figure 2:**
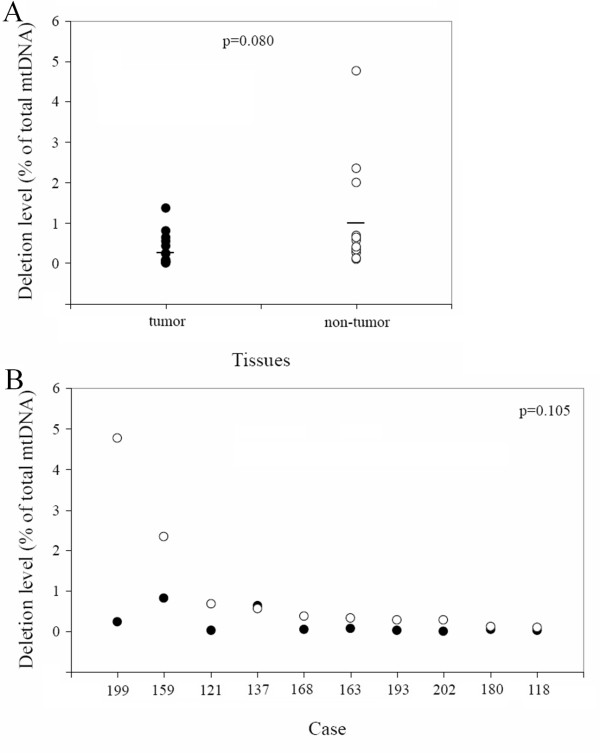
**Comparison of mtDNA 4,977-bp deletion levels between tumor and non-tumor tissues**. (A) deletion levels in 17 and 13 patients carrying the deletion in tumor (closed circle and solid line)and non-tumor tissues(open circle), respectively. The solid lines represent the mean values. (B) paired deletion levels in tumor and non-tumor tissues from the same patients in 10 cases who carried the deletion in both tissues. p-values were obtained by independent-samples T test for (A) and paired-samples T test for (B).

We then measured levels of the 4,977-bp mtDNA deletions in the patients carrying it. We found that the content of the mtDNA deletion varied from 0.0109% to 4.77% of total mitochondrial DNA in tissues carrying this deletion. Interestingly, although failed to achieve the significance, the average and mean levels of deletion in 17 tumor tissues appeared lower than those in non-tumor areas (Figure. 2A). Moreover, in patients who carried the deletion in both tumor and nearby non-tumor tissues, the deletion levels were almost always lower in tumor tissues compared with the non-tumor areas (Figure. 2B). Interestingly, the P-value was 0.047 when the outlier case 199 was excluded in analysis. These results indicate a negative selection for the 4,977-bp deletion in cancer cells.

To further explore the role of the 4,977-bp deletion in the pathogenesis of colorectal cancer, we examined the relationship between deletion level and some risk factors or clinicopathologic features in patients carrying this deletion in tumor tissues or non-tumor tissues. Interestingly, we found after multiple linear regression analysis that the deletion level in tumor tissues decreased as the cancer stage advanced (p = 0.031) (Table 2). No correlations were found between deletion levels in tumor or non-tumor tissues with age, BMI, differentiation or LN metastasis.

**Table 2:** Multiple linear regression analysis of the relationship between mtDNA 4,977 bp deletion level and age, BMI, metastasis status and stage of cancer in tumor and non-tumor tissues in 20 patients with the deletion.

Deletion level	Characteristics	Coefficients (B)	95% CI for B	p-value
Non-tumor	Age	0.000	-0.002 to 0.001	0.245
	BMI	0.000	-0.003 to 0.004	0.859
	LN metastasis	-0.023	-0.074 to 0.029	0.337
	Stage	-0.011	-0.043 to 0.020	0.415
Tumor	Age	0.000	0.000 to 0.000	0.141
	BMI	0.000	0.000 to 0.001	0.471
	LN metastasis	-0.006	-0.014 to 0.002	0.115
	Stage	-0.005	-0.010 to 0.000	0.031*

BMI, body mass index; LN metastasis, lymph node metastasis; Stage, tumor-node-metastasis (TNM) stage; Coefficients: the effect of the corresponding characteristics on deletion level; 95% CI, 95% confidence interval; * statistically significant.

### The 4,977 bp deletion level and mtDNA copy number in colorectal cancer patients

**Figure 3:**
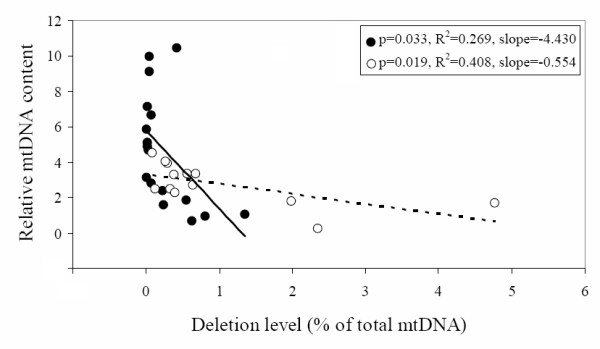
**Relationship between mtDNA content and the 4,977-bp deletion level**. The deletion level in tumor (closed circle and solid line) and non-tumor (open circle and dot line) tissues from 20 patients who carried the deletion are plotted against the mtDNA content. R^2 ^indicates the strength of a linear relationship between the two parameters.

Alterations in mtDNA content have been reported in increasing numbers of cancer types [28]. To investigate mtDNA copy number changes and to determine if there was a correlation between the common deletion and the overall mtDNA content in colorectal cancer, we further analyzed mtDNA copy numbers in these colorectal cancer patients. We first plotted the mtDNA copy numbers vs. 4,977 bp deletion levels in tumor and non-tumor tissues of all 20 colorectal cancer patients carrying this deletion. Surprisingly, we found that, with the decline of the common deletion, mtDNA copy number increased in both tumor and non-tumor tissues (Figure. 3). Nevertheless, the slope of increase of mtDNA copy number with decrease of common deletion level in tumor tissues were much steeper compared with nearby non-tumor areas, indicating a stronger effect of the cancer nuclear background on modulating the mtDNA content in presence of common deletion.

**Figure 4:**
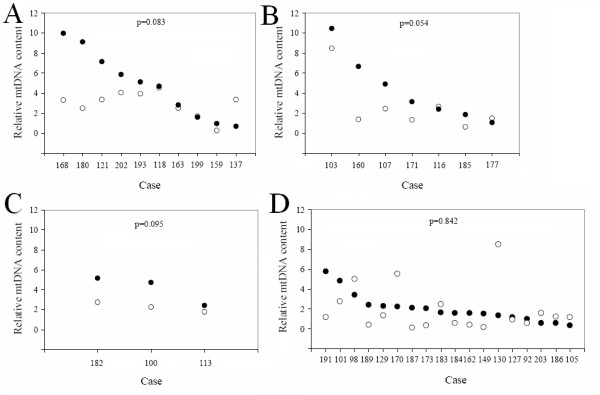
**Comparison of mtDNA content between tumor and non-tumor tissues**. mtDNA content in tumor (closed circle) and non-tumor (open circle) tissues are compared in patients (A) carrying the mtDNA 4,977-bp deletion in both tissues (B) carrying the mtDNA 4,977-bp deletion only in tumor tissues, (C) carrying the mtDNA 4,977-bp deletion only in non-tumor tissues, and (D) age and gender matched patients without the deletion. All p values were derived from paired-samples T test analysis.

To verify the difference in mtDNA copy number changes in tumors and nearby non-tumor tissues, we compared them in patients carrying the 4,977-bp deletion in both tumor and non-tumor areas (Figure. 4A), and in patients carrying this deletion only in tumor tissues (Figure. 4B) or only in non-tumor tissues (Figure. 4C). In almost all cases, we found that mtDNA copy numbers were higher in tumor tissues compared with the nearby non tumor areas. To further determine if the 4,977-bp deletion was implicated in the alteration of mtDNA content in colorectal cancer, we also analyzed the mtDNA copy numbers in 18 age- and gender-matched control colorectal cancer patients who did not carry the 4,977-bp deletion. As shown in Figure. 4D, unlike what we observed in patients with the deletion, we did not see a consistent difference in mtDNA copy numbers between tumor and non tumor tissues.

**Figure 5:**
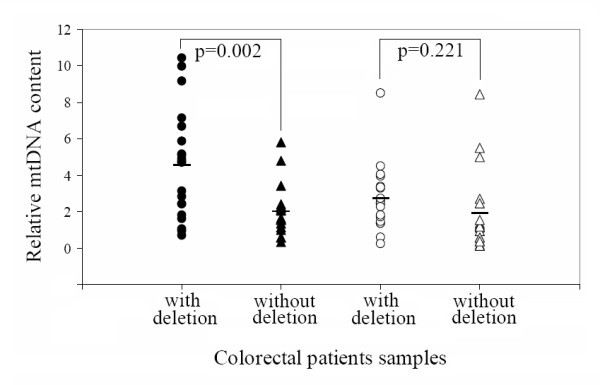
**Comparison of mtDNA content between patients with and without the 4, 977-bp mtDNA deletion**. mtDNA contents in tumor and non-tumor tissues from 20 patients with 4,977 bp deletion and 18 age- and gender-matched patients without the deletion were compared (tumor, closed circle/triangle; non-tumor, open circle/triangle; circle, with common deletion; triangle, without common deletion). The solid lines represent the mean values. All p values were derived from independent-samples T test analysis.

We further compared mtDNA copy number in tumor and non-tumor tissues from patients with and without the mtDNA 4,977-bp deletion. As shown in Figure. 5, while there was a significant difference between the mtDNA copy numbers in non-tumor tissues between colorectal cancer patients with or without the 4,977-bp deletion (20 with and 18 without, age- and gender-matched patients), mtDNA content in the tumor tissues was higher in patients with the mtDNA common deletion (p = 0.002).

### mtDNA copy number as a potential marker in colorectal cancer

**Figure 6:**
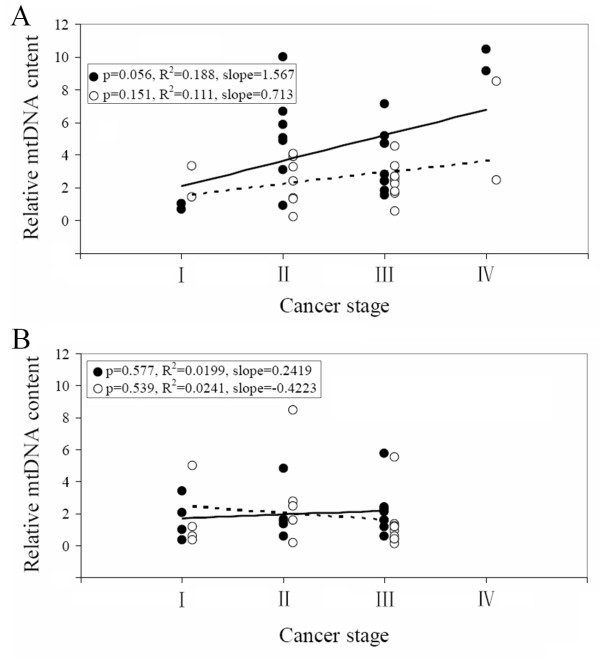
**Relationship between mtDNA content and cancer stage**. mtDNA content is plotted again the cancer stage in tumor (closed circle) and non-tumor (open circle) tissues of patients (A) with the 4, 977-bp mtDNA deletion, and (B) without the deletion.

To further determine if the mtDNA copy number could also serve as a biomarker for development of colorectal cancer, we analyzed the relationship between mtDNA copy number and cancer stage in these patients. Since we showed previously that in patients with the common deletion, cancer stage was correlated with the 4,977-bp deletion level (Table 2), we examined the relationship between mtDNA content and cancer stage in patients with and without the common deletion. As shown in Figure. 6, in patients with the 4,977-bp deletion, it appeared that as the cancer stage advanced, the mtDNA copy number increased (p = 0.056) in the tumors, while no such correlation was observed in the nearby non-tumor areas (p = 0.151) (Figure. 6A). The correlation between mtDNA content in tumor tissues and cancer stage became stronger and significant after multiple linear regression analysis (p < 0.001) (Table 3). However, in the 18 age- and gender-matched patients without the common deletion, no correlations were detected in both tumor and non-tumor tissues (Figure. 6B and Table 3). To further investigate the implication of mtDNA common deletion and mtDNA content in colorectal cancer patients, we carried out a multiple linear regression analysis of relationship between mtDNA content and age, metastasis status and cancer stage in tumor and non-tumor tissues in patients with or without the common deletion. As shown in Table 3, significant correlations only existed in tumor tissues of colorectal cancer patients carrying the mtDNA common deletion. Besides the cancer stage, with increasing mtDNA copy number in tumor tissues with the common deletion, the cancer was more likely to be LN metastatic (p = 0.002).

**Table 3:** Multiple linear regression analysis of relationships between mtDNA content and age, metastasis status and stage of cancer in tumor and non-tumor tissues in patients with or without the 4,977 bp mtDNA deletion

**mtDNA content**		**Patients without 4977 bp deletion**			**Patients with 4977 bp deletion**		
		
	**Characteristics**	**Coefficients (B)**	**95% CI for B**	**p-value**	**Coefficients (B)**	**95% CI for B**	**p-value**
Non-tumor	Age	0.191	-0.220 to 0.602	0.333	0.027	-0.055 to 0.108	0.493
	LN metastasis	25.029	-0.328 to 50.386	0.053	2.137	-1.464 to 5.739	0.224
	Stage	14.460	-1.759 to 30.679	0.076	1.690	-0.614 to 3.993	0.138
Tumor	Age	0.017	-0.052 to 0.087	0.602	0.064	-0.033 to 0.161	0.180
	LN metastasis	-0.330	-4.619 to 3.959	0.871	7.880	3.578 to 12.182	0.002*
	Stage	-0.100	-2.844 to 2.643	0.938	5.839	3.088 to 8.590	0.000*

LN metastasis, lymph node metastasis; Stage, tumor-node-metastasis (TNM) stage; Coefficients: effect of the corresponding characteristics on deletion level; 95% CI, 95% confidence interval; * statistically significant.

## Discussion

mtDNA mutations have been implicated in various human diseases including cancer [24], a long-term process that involves multiple steps driven by different genetic and epigenetic alterations. Among the mtDNA mutations, the 4,977-bp deletion is one of the most frequent [23]. Several studies have found the mtDNA 4,977-bp deletion in various types of cancer, including in cancer of the breast, endometrial, esophagus, stomach, head and neck, liver, lung, mouth, kidney, skin and thyroid [24,28]. However, in some cases, the incidence and level of the 4,977 bp deletion were lower in the tumor tissues compared with nearby non-tumor tissues from the same patients[29]. Thus, the role of this common deletion in tumorigenesis is intriguing, but largely perplexing.

In our first finding, unlike the age-dependent accumulation that was expected, the 4,977 bp deletion was detected more frequently in tumor tissues of patients younger than 65 (12/48, or 25%) compared to patients over 65 (5/56, or 8.9%) (p = 0.027). This result indicated that there is possibly a negative selection for the common deletion in tumor tissues during aging. In addition, as previously reported with thyroid, renal and liver cancer patients [29], we found the deletion level in tumor tissues was likely to be lower than that in the nearby non-tumor areas. In particular, in 10 patients carrying the 4,977-bp deletion in both tumor and nearby non-tumor tissues, the deletion levels were almost all lower in tumor tissues, indicating a negative selection for the common deletion in the cancer cells. It is also interesting to note that among these 10 subjects, patient 199 was at an advanced tumor stage and exhibited metastatic features, and showed the biggest difference in the deletion levels between tumor and non-tumor tissues and the highest level of the common deletion in the non-tumor areas (Figure. 2B). On the other hand, patient 137 exhibited very similar deletion level in tumor tissues and the nearby non-tumor tissues (Figure. 2B), and he was at the early stage of cancer and exhibited no metastasis. Furthermore, among all 17 patients who carried the deletion in tumor tissues, there was a good correlation after multiple linear regression analysis (p = 0.031) between the decrease in deletion level in tumor tissues and the stage of advancement in the cancer.

To explain the observed results, we hypothesize that, as we previously found in tumorigenesis studies on cells carrying mtDNA with heteroplasmic and homoplasmic mutations in the complex I subunit ND5 gene [30], the 4,977-bp mtDNA deletion could function in cancer development as follows: in the initial stage, when cancer cells are under stress because of a carcinogenic insult or oxidative stress damage, the deletion emerges. Because of the replicative advantage of smaller mtDNA, mtDNA with the 4,977-bp deletion is enriched to a certain level which would enhance tumor progression due to retrograde pathways [31]. Retrograde regulation is a communication pathway from mitochondria to the nucleus, and is usually used to describe the cellular responses to changes in the functional state of mitochondria. One of the mechanisms suggested to play a role in the retrograde response was mitochondrial stress, which is supported by changes in mitochondrial membrane potential and calcium elevation [31]. However, at certain stage of tumorigenesis, it may become more important to have a functional respiratory chain than an inhibited one to sustain rapid cell proliferation. As a result, compared with the nearby non-tumor tissues, mtDNA with the common deletion becomes diluted out in tumor tissues.

Low mtDNA content has been reported to be associated with increased risk of renal cancer carcinoma [32], and a decrease in mtDNA copy number in cancer tissues has been found also in gastric cancer [33], breast cancer [34] and hepatocellular carcinoma [35]. On the other hand, an increase in mtDNA content was reported in the majority of renal oncocytomas [36], head and neck cancer [37], endometrial cancer [38], ovarian cancer [39] and colorectal cancer [40]. It is therefore suggested that the change in mtDNA content is cancer type specific [28,34]. However the underlying molecular mechanisms of alteration in mtDNA in cancer cells are largely unclear.

Our results strongly suggest that the 4,977-bp deletion is important in the specific up-regulation of mtDNA content in tumor tissues. The mtDNA content in tumor tissue of almost all of 20 patients who carried the 4,977-bp deletion was higher than that in the nearby non-tumor areas. The only exception was patient 137 (Figure. 4A), who was in her early stage of cancer and without detection of metastasis in the lymph nodes. On the other hand, patient 180 (Figure. 4A), who showed the biggest difference in the levels of mtDNA content between tumor and non-tumor areas, was in the terminal stage of cancer and with metastasized lymph nodes. These results support the notion that tumor background favors high mtDNA copy number with the presence of the 4,977-bp deletion. As this deletion removes all or part of the genes encoding four complex I subunits, one complex IV subunit, two complex V subunits and five tRNA genes, which are indispensable for maintaining normal mitochondrial function [41], the mtDNA 4,977-bp deletion could lead to energy production catastrophes [42] and abnormal ROS generation [43]. Since the deletion levels observed here were well below the threshold for any bioenergetics consequences [42], the up-regulation is more likely due to a retrograde reaction [31] rather than a simple compensatory effect for ATP production. This retrograde signal is amplified in a colorectal cancer background.

## Conclusions

In conclusion, our investigation provides evidence that the mtDNA 4,977-bp deletion plays a role in the early stage of colorectal cancer, but it is selected against once the tumor enter the rapid growth phase. Our results also demonstrate that in tumors with this common deletion, mtDNA content could increase specifically in tumor tissues, probably due to a retrograde effect. These results also indicate that both the 4,977-bp deletion and mtDNA content may serve as a biomarker for colorectal cancer in some patients.

## Competing interests

The authors declare that they have no competing interests.

## Authors' contributions

TC carried out the mtDNA analysis. TC and JH carried out the statistical analysis. TC, LS, HF, HN, TJ, XW, YX, YJ and GC collected samples and carried out the pathological analysis. YB, JL and TC conceived the study, participated in the design the experiments and drafted the manuscript. All authors read and approved the final manuscript.

## Pre-publication history

The pre-publication history for this paper can be accessed here:

http://www.biomedcentral.com/1471-2350/12/8/prepub

## References

[B1] Jemal A Cancer statistics, 2009 CA Cancer J Clin 2009 59 4 225 49 10.3322/caac.20006 19474385

[B2] Sung JJ Asia Pacific consensus recommendations for colorectal cancer screening Gut 2008 57 8 1166 76 10.1136/gut.2007.146316 18628378

[B3] Lei T Prevalence trend of colorectal cancer in 10 cities and counties in China from 1988 to 2002 Zhonghua Zhong Liu Za Zhi 2009 31 6 428 33 19950551

[B4] Lichtenstein P Environmental and heritable factors in the causation of cancer--analyses of cohorts of twins from Sweden, Denmark, and Finland N Engl J Med 2000 343 2 78 85 10.1056/NEJM200007133430201 10891514

[B5] Czene K Lichtenstein P Hemminki K Environmental and heritable causes of cancer among 9.6 million individuals in the Swedish Family-Cancer Database Int J Cancer 2002 99 2 260 6 10.1002/ijc.10332 11979442

[B6] Dionigi G Genetic alteration in hereditary colorectal cancer Surg Oncol 2007 16 Suppl 1 S11 5 10.1016/j.suronc.2007.10.020 18023570

[B7] Walther A Genetic prognostic and predictive markers in colorectal cancer Nat Rev Cancer 2009 9 7 489 99 10.1038/nrc2645 19536109

[B8] Wallace DC Mitochondria as chi Genetics 2008 179 2 727 35 10.1534/genetics.104.91769 18558648 PMC2429869

[B9] Fang H Cancer type-specific modulation of mitochondrial haplogroups in breast, colorectal and thyroid cancer BMC Cancer 2010 10 421 30 10.1186/1471-2407-10-421 20704735 PMC2933623

[B10] Warburg O On respiratory impairment in cancer cells Science 1956 124 3215 269 70 13351639

[B11] Schon EA Mitochondrial genetics and disease Trends Biochem Sci 2000 25 11 555 60 10.1016/S0968-0004(00)01688-1 11084368

[B12] Schon EA Bonilla E DiMauro S Mitochondrial DNA mutations and pathogenesis J Bioenerg Biomembr 1997 29 2 131 49 10.1023/A:1022685929755 9239539

[B13] Wallace DC Mitochondrial DNA mutations in human degenerative diseases and aging Biochim Biophys Acta 1995 1271 1 141 51 7599200 10.1016/0925-4439(95)00021-u

[B14] Shen L Evaluating mitochondrial DNA in patients with breast cancer and benign breast disease J Cancer Res Clin Oncol10.1007/s00432-010-0912-x PMC11827960 20552226

[B15] Clay Montier LL Deng JJ Bai Y Number matters: control of mammalian mitochondrial DNA copy number J Genet Genomics 2009 36 3 125 31 10.1016/S1673-8527(08)60099-5 19302968 PMC4706993

[B16] Brandon M Baldi P Wallace DC Mitochondrial mutations in cancer Oncogene 2006 25 34 4647 62 10.1038/sj.onc.1209607 16892079

[B17] Chatterjee A Mambo E Sidransky D Mitochondrial DNA mutations in human cancer Oncogene 2006 25 34 4663 74 10.1038/sj.onc.1209604 16892080

[B18] Shen L Evaluating mitochondrial DNA in cancer occurrence and development Ann N Y Acad Sci 1201 26 33 10.1111/j.1749-6632.2010.05635.x 20649535

[B19] Lezza AM Mitochondrial DNA 4977 bp deletion and OH8dG levels correlate in the brain of aged subjects but not Alzheimer's disease patients Faseb J 1999 13 9 1083 8 10336891 10.1096/fasebj.13.9.1083

[B20] Holt IJ Harding AE Morgan-Hughes JA Deletions of muscle mitochondrial DNA in patients with mitochondrial myopathies Nature 1988 331 6158 717 9 10.1038/331717a0 2830540

[B21] Zeviani M Deletions of mitochondrial DNA in Kearns-Sayre syndrome Neurology 1988 38 9 1339 46 3412580 10.1212/wnl.38.9.1339

[B22] Taylor RW Turnbull DM Mitochondrial DNA mutations in human disease Nat Rev Genet 2005 6 5 389 402 10.1038/nrg1606 15861210 PMC1762815

[B23] Meissner C The 4977 bp deletion of mitochondrial DNA in human skeletal muscle, heart and different areas of the brain: a useful biomarker or more? Exp Gerontol 2008 43 7 645 52 10.1016/j.exger.2008.03.004 18439778

[B24] Lu J Sharma LK Bai Y Implications of mitochondrial DNA mutations and mitochondrial dysfunction in tumorigenesis Cell Res 2009 19 7 802 15 10.1038/cr.2009.69 19532122 PMC4710094

[B25] Polyak K Somatic mutations of the mitochondrial genome in human colorectal tumours Nat Genet 1998 20 3 291 3 10.1038/3108 9806551

[B26] Lievre A Clinical value of mitochondrial mutations in colorectal cancer J Clin Oncol 2005 23 15 3517 25 10.1200/JCO.2005.07.044 15908662

[B27] Ding Z Analysis of mitochondrial DNA mutations in D-loop region in thyroid lesions Biochim Biophys Acta 1800 3 271 4 19463899 10.1016/j.bbagen.2009.05.009

[B28] Lee HC Wei YH Mitochondrial DNA instability and metabolic shift in human cancers Int J Mol Sci 2009 10 2 674 701 10.3390/ijms10020674 19333428 PMC2660656

[B29] Dani SU Dani MA Simpson AJ The common mitochondrial DNA deletion deltamtDNA(4977): shedding new light to the concept of a tumor suppressor mutation Med Hypotheses 2003 61 1 60 3 10.1016/S0306-9877(03)00105-1 12781642

[B30] Park JS A heteroplasmic, not homoplasmic, mitochondrial DNA mutation promotes tumorigenesis via alteration in reactive oxygen species generation and apoptosis Hum Mol Genet 2009 18 9 1578 89 10.1093/hmg/ddp069 19208652 PMC2733816

[B31] Butow RA Avadhani NG Mitochondrial signaling: the retrograde response Mol Cell 2004 14 1 1 15 10.1016/S1097-2765(04)00179-0 15068799

[B32] Xing ea Mitochondrial DNA Content: Its Genetic Heritability and Association With Renal Cell Carcinoma J. Natl. Cancer Inst 2008 100 15 1104 1112 10.1093/jnci/djn213 18664653 PMC2720693

[B33] Wu CW Mitochondrial DNA mutations and mitochondrial DNA depletion in gastric cancer Genes Chromosomes Cancer 2005 44 1 19 28 10.1002/gcc.20213 15892105

[B34] Mambo E Tumor-specific changes in mtDNA content in human cancer Int J Cancer 2005 116 6 920 4 10.1002/ijc.21110 15856456

[B35] Yin PH Alteration of the copy number and deletion of mitochondrial DNA in human hepatocellular carcinoma Br J Cancer 2004 90 12 2390 6 15150555 10.1038/sj.bjc.6601838 PMC2409531

[B36] Heddi A Coordinate expression of nuclear and mitochondrial genes involved in energy production in carcinoma and oncocytoma Biochim Biophys Acta 1996 1316 3 203 9 8781539 10.1016/0925-4439(96)00026-9

[B37] Kim MM Mitochondrial DNA quantity increases with histopathologic grade in premalignant and malignant head and neck lesions Clin Cancer Res 2004 10 24 8512 5 10.1158/1078-0432.CCR-04-0734 15623632

[B38] Wang Y The increase of mitochondrial DNA content in endometrial adenocarcinoma cells: a quantitative study using laser-captured microdissected tissues Gynecol Oncol 2005 98 1 104 10 10.1016/j.ygyno.2005.04.015 15921730

[B39] Wang Y Association of decreased mitochondrial DNA content with ovarian cancer progression Br J Cancer 2006 95 8 1087 91 10.1038/sj.bjc.6603377 17047655 PMC2360719

[B40] Lee HC Mitochondrial genome instability and mtDNA depletion in human cancers Ann N Y Acad Sci 2005 1042 109 22 10.1196/annals.1338.011 15965052

[B41] Shoffner JM Spontaneous Kearns-Sayre/chronic external ophthalmoplegia plus syndrome associated with a mitochondrial DNA deletion: a slip-replication model and metabolic therapy Proc Natl Acad Sci USA 1989 86 20 7952 6 10.1073/pnas.86.20.7952 2554297 PMC298190

[B42] Porteous WK Bioenergetic consequences of accumulating the common 4977-bp mitochondrial DNA deletion Eur J Biochem 1998 257 1 192 201 10.1046/j.1432-1327.1998.2570192.x 9799119

[B43] Peng TI Visualizing common deletion of mitochondrial DNA-augmented mitochondrial reactive oxygen species generation and apoptosis upon oxidative stress Biochim Biophys Acta 2006 1762 2 241 55 16368227 10.1016/j.bbadis.2005.10.008

